# Synthesis, characterization, and DNA binding of Ni(II), Cu(II), and Zn(II) complexes with a novel N,O-bidentate acylhydrazone

**DOI:** 10.1007/s10534-026-00856-0

**Published:** 2026-07-18

**Authors:** Aujenus Albert Msumange, Jeniffer Meyer Moreira, Deus Albert Msumange, Rafael Aparecido Carvalho Souza, Mário Machado Martins, Cristiane Storck Schwalm, Luana da Silva Dorneles, Magno Aparecido Gonçalves Trindade, Raphael Rodrigues, Eduardo José de Arruda, Cláudio Teodoro de Carvalho

**Affiliations:** 1 Federal University of Grande Dourados - Quality Control and Thermal Analysis Laboratory (LabCAT), Dourados, MS 79804-970 Brazil; 2 Centre for Advanced Materials and Industrial Chemistry (CAMIC), School of Science, RMIT University, Melbourne, VIC 3000 Australia; 3 Federal University of Uberlândia, Uberlândia, MG 38400-902 Brazil

**Keywords:** Novel Schiff base complexes, TGA–DSC, DNA interaction, Molecular docking

## Abstract

A novel acylhydrazone Schiff base ligand, 2-fluoro-N′-[(1E,2E)-3-(2-methoxyphenyl)prop-2-en-1-ylidene]benzohydrazide (L), was synthesized and coordinated with Ni(II), Cu(II), and Zn(II) ions. The resulting complexes were characterized using TGA–DSC, FTIR/ATR, HRESIMS, ^1^H NMR, PXRD, elemental analysis, and complexometric titration, confirming a 1:2 metal-to-ligand stoichiometry and the formation of polycrystalline compounds. Coordination occurs via an N,O-bidentate mode through the enolic oxygen and azomethine nitrogen, forming stable five-membered chelate rings. Thermal analysis revealed high stability, with decomposition leading to the metal oxides, while PXRD indicated crystallite sizes in the 20–50 nm range. Spectroscopic DNA-binding studies via UV–Vis and fluorescence spectroscopy demonstrated moderate hypochromism (20–41%) without significant bathochromic shifts, yielding intrinsic binding constants (K_b_) on the order of 10^4^ L mol^−1^. Viscosity measurements and ethidium bromide displacement assays corroborated a predominantly non-intercalative groove-binding mode. Molecular docking simulations supported these experimental findings, revealing plausible minor-groove binding poses stabilized by hydrogen bonding, halogen contacts, π–anion interactions, and hydrophobic interactions, consistent with the observed DNA-binding behavior. BSA fluorescence quenching studies suggested a predominant static quenching mechanism, with binding constants in the range of 10^4^–10^5^ M^−1^, indicative of moderate, reversible ground-state complex formation with serum albumin and suggesting relevant protein-binding behavior. The combined experimental and theoretical data demonstrate that metal coordination enhances DNA affinity and modulates biomolecular interactions, particularly for the Ni(II) complex, highlighting the potential of this new acylhydrazone framework as a platform for DNA-binding coordination compounds and related studies of biomolecular interactions.

**Supplementary Information:**

The online version contains supplementary material available at 10.1007/s10534-026-00856-0.

## Introduction

Schiff bases are widely studied because of their structural flexibility, ease of synthesis, and broad spectrum of applications in medicinal, catalytic, analytical, and material sciences. In recent years, Schiff base metal complexes have gained increasing attention as promising candidates in medicinal chemistry due to their enhanced biological activities, including antioxidant, antibacterial, antifungal, anti-inflammatory, and anticancer properties. These complexes have also shown significant potential in DNA/BSA interactions, catalysis, chemical sensing, corrosion inhibition, and optical materials. The coordination of Schiff bases with metal ions often yields stable complexes with diverse geometries and enhanced physicochemical and biological properties, making them highly relevant in modern bioinorganic and pharmaceutical research. Their chemistry mainly centers on the azomethine/imine group (–C=N–), formed through a simple condensation reaction between a primary amine and an aldehyde or ketone. This synthetic flexibility enables the design of a wide variety of functional ligands and metal complexes with tunable properties (Jiang et al. 2024; Thakor et al. 2023; Zengin et al. 2023).

Within the broad family of Schiff bases**,** N-acylhydrazones (R₁–NHN=CH–R₂) have become particularly important in different biological applications because of their structural flexibility and role as potential pharmacophores. Their strong tendency to coordinate with a variety of metal ions makes them candidates for forming more stable coordination complexes. Beyond their structural versatility, acylhydrazones display an impressive spectrum of biological effects, ranging from antimicrobial (Dinku et al. 2024; Moreno-Narváez et al. 2025; Rathi et al. 2026), anti-inflammatory (Kajal et al. 2014; Medeiros et al. 2021), anticancer (Biliz et al. 2023; Fekri et al. 2022; Gholamrezazadeh and Kohanfekr 2026; Tyagi and Dubey 2025), antifungal (Luitel et al. 2026; Tang et al. 2023), antibacterial (Dhanya et al. 2025; Lin et al. 2022; Qiao et al. 2025), and antiviral properties (Cui et al. 2024). Their ability to engage with key biological macromolecules, most notably DNA, highlights their significance as promising scaffolds for targeted therapeutic development.

In recent years, considerable attention has been devoted to first-row transition metal complexes because of their diverse coordination chemistry, redox properties, and wide range of biological and technological applications. These elements are not only abundant and relatively less toxic than heavier metals, but they also play essential roles in many biological systems, as reported in various studies (Mali et al. 2026; Wang et al. 2021). The ability of various metals to switch between oxidation states and form stable coordination environments with diverse donor atoms makes them attractive research targets. Consequently, they have become central to studies in medicinal chemistry, catalysis, and bioinorganic chemistry, where their interactions with DNA, proteins, and other biomolecules hold significant promise for therapeutic development and technological applications (Khor et al. 2020; Marinova and Tamahkyarova 2025; Rajan Arun et al. 2024).

Because transition metals and their corresponding ligands play essential roles in biological and chemical systems, understanding their interactions with DNA is crucial for exploring potential biomedical applications. DNA is a primary intracellular target and provides several binding sites for small molecules, enabling diverse interaction modes such as intercalation between base pairs, groove binding within the major and minor grooves, and electrostatic interactions with the negatively charged phosphate backbone (Kumar et al. 2023; McQuaid et al. 2022; Triviño-Rojas et al. 2025). Although Schiff base metal complexes have been extensively investigated for their DNA-binding properties, there remains a growing need to develop new molecular architectures with enhanced binding strength and selectivity, ultimately enabling more effective applications in medicinal chemistry and related fields.

Based on our previous work on metal-based hydrazone systems, we identified an opportunity to explore new molecular architectures, as the Schiff base ligand 2-fluoro-N′-[(1E,2E)-3-(2-methoxyphenyl)prop-2-en-1-ylidene]benzohydrazide has not been reported in the literature to date. A comprehensive literature survey was carried out using multiple scientific databases, including SciFinder, Reaxys, Web of Science, Scopus, and other relevant search platforms. The search revealed no prior reports of this ligand or its corresponding Ni(II), Cu(II), and Zn(II) complexes in the available literature; its synthesis, structural characterization, and biological properties, both in free and metal-complexed forms, remain unexplored. Considering that even minor structural modifications can significantly influence biological activity, the ligand was synthesized from 2-fluorobenzohydrazide and (2E)-3-(2-methoxyphenyl)prop-2-enal. Subsequently, its complexes with biologically relevant Ni(II), Cu(II), and Zn(II) ions were prepared. The ligand and its complexes were characterized using ^1^H NMR, FTIR/ATR, UV–Vis spectroscopy, PXRD, TGA–DSC, HRESIMS, elemental analysis, and complexometric titration. Furthermore, their DNA and BSA binding properties were investigated using spectroscopic titrations and viscosity measurements, and the experimental findings were supported by molecular docking studies to elucidate their interaction modes.

## Materials and methods

All solvents and reagents used in this study were commercially obtained and used without further purification. Calf thymus DNA (CT-DNA, Type I, D-1501) and bovine serum albumin (BSA, A-2153) employed in the biomolecular interaction studies were purchased from Sigma-Aldrich (St. Louis, MO, USA).

The precursors for the Schiff base ligand, 2-fluorobenzohydrazide (97%) and (2E)-3-(2-methoxyphenyl)prop-2-enal (2-methoxycinnamaldehyde, 98%), were purchased from the same supplier. Nickel (II) chloride hexahydrate (NiCl₂·6H₂O), copper (II) sulfate pentahydrate (CuSO₄·5H₂O), zinc chloride (ZnCl₂), methanol (99.9%), and sodium bicarbonate (NaHCO₃, 99.7%) were purchased from commercial suppliers. Triethylamine (99%) was obtained from Êxodo Científica (Sumaré, SP, Brazil).

### Synthesis of the ligand

**Scheme 1:**
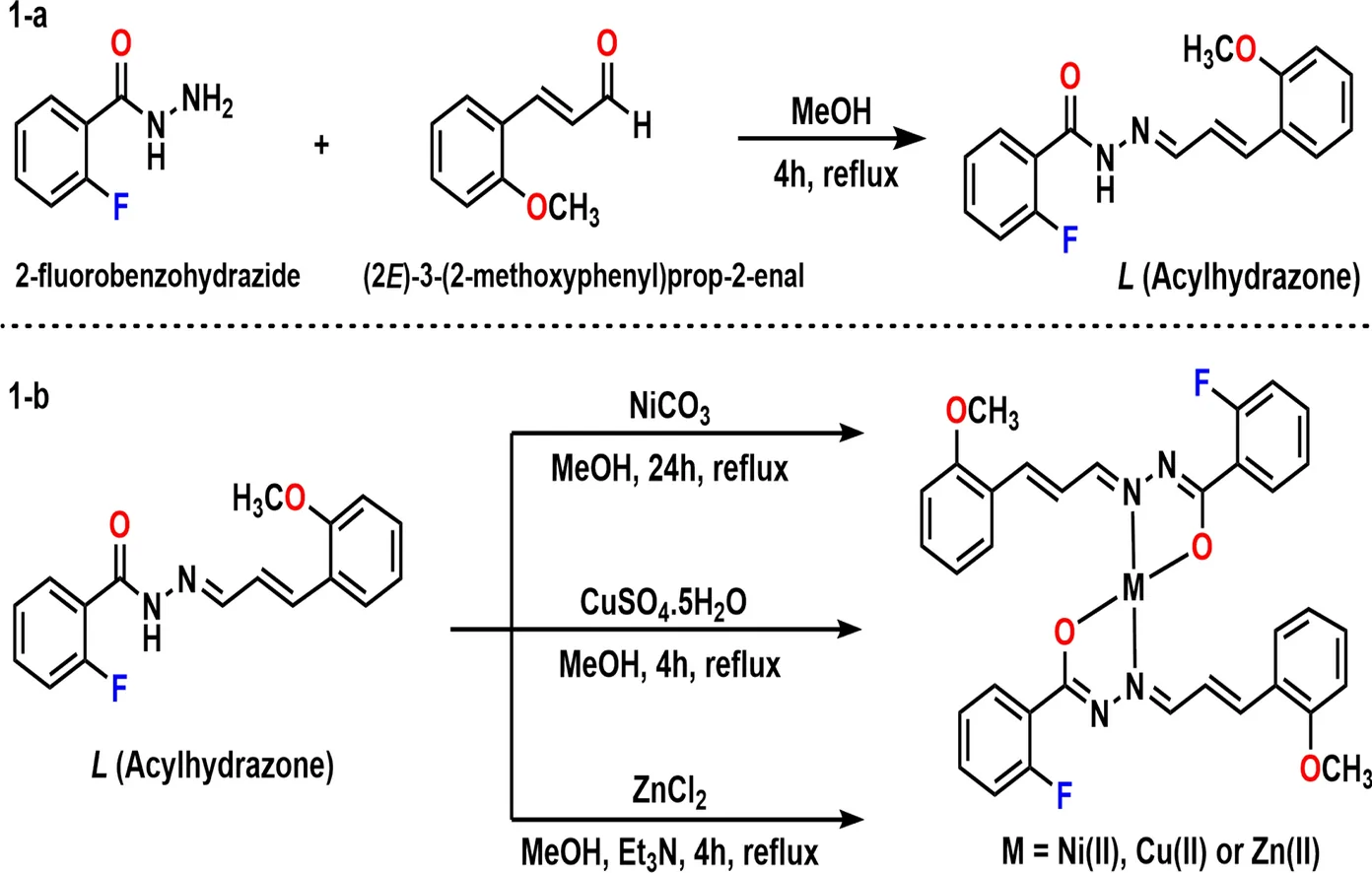
**a** and **b** Simplified representation of the synthetic route and the proposed coordination mode for the ligand and its Ni(II), Cu(II), and Zn(II) complexes

Ligand synthesis (Scheme 1a). The ligand was prepared by dissolving 0.661 g (4.288 mmol) of 2-fluorobenzohydrazide (154.14 g/mol) in 10 mL of methanol in a 50 mL round-bottom flask. To this solution, 0.696 g (4.291 mmol) of (2E)-3-(2-methoxyphenyl)prop-2-enal (162.19 g/mol) dissolved in 5 mL of methanol was added dropwise under constant stirring. The reaction was carried out using an equimolar stoichiometric ratio (1:1) of hydrazide to aldehyde. After completion of the reaction, the mixture was filtered through Whatman No. 40 filter paper, and the resulting solid was washed with methanol and dried in an oven at 50 °C for 24 h. The dried product was weighed, giving a 90% yield, and stored in a desiccator for further analysis.

The molecular formula of the acylhydrazone ligand 2-fluoro-N′-[(1E,2E)-3-(2-methoxyphenyl)prop-2-en-1-ylidene]benzohydrazide(C_17_H_15_FN_2_O_2_, M = 298.11 gmol^−1^), was confirmed by elemental analysis (EA). Found (%): (C = 68.2%, H = 5.01%, N = 9.28%). The purity was estimated at 99.9% by DSC (Fig. S1). Comparison of the ^1^HNMR spectra of the free ligand and its zinc complex, consistent with an N,O-bidentate coordination mode, is shown in Figure S2, which is representative of the other complexes.

### Synthesis of the complexes

Similar to the ligand synthesis, the preparation of the complexes was carried out by adapting procedures previously reported in the literature, as described by Moreira et al. (2025).

Ni(II) complex: The synthesis of the nickel complex (Scheme 1b) was carried out by reacting the ligand and NiCO₃ in a stoichiometric 2:1 molar ratio. Thus, 0.146 g (0.4898 mmol) of the ligand was dissolved in 25 mL of methanol in a 50 mL round-bottom flask under magnetic stirring and heating. Subsequently, a 2 mL methanolic solution of 0.029 g (0.2443 mmol) of synthesized NiCO₃ was added dropwise. The procedure for synthesizing the metallic carbonate follows the methodology reported by Caires et al. (2013). The reaction mixture was maintained under reflux (60 °C) and magnetic stirring for 24 h to allow complete reaction and removal of carbon dioxide gas. The precipitate was then filtered and washed with methanol. A qualitative test with dilute hydrochloric acid was performed on the resulting precipitate to detect any gas bubbles, which would indicate the presence of unreacted carbonate. No effervescence was observed, confirming that the carbonate had fully reacted. The resulting product was obtained as a powder with a 47% yield. Found (%) by EA: C, 62.01; H, 4.231; N, 8.601.

Cu(II) complex: The synthesis of the copper complex (Scheme 1b) was carried out using a slight excess of ligand relative to the metal source. Thus, 0.167 g (0.5601 mmol) of the ligand was dissolved in 25 mL of methanol in a 50 mL round-bottom flask under magnetic stirring and heating. Subsequently, a 2 mL methanolic solution containing 0.063 g (0.2524 mmol) of CuSO₄·5H₂O was added dropwise. The reaction mixture was maintained under reflux (60 °C) and magnetic stirring for 4 h. After cooling, the precipitate was filtered, then washed with methanol and dried for further analysis. The resulting product was obtained as a powder with a yield of 56%. Found (%) by EA: C, 61.80; H, 4.110; N, 8.481.

Zn(II) complex: The zinc complex (Scheme 1b) was synthesized using the ligand and zinc salt in a stoichiometric 2:1 molar ratio. Thus, 0.108 g (0.3623 mmol) of the ligand was dissolved in 25 mL of methanol in a 50 mL round-bottom flask with magnetic stirring and gentle heating. A methanolic solution of ZnCl₂ (0.025 g, 0.1834 mmol, 2 mL) was then added dropwise, followed by the addition of five drops of triethylamine. The reaction mixture was refluxed at 60 °C under continuous stirring for 4 h. After cooling, the resulting pale-yellow precipitate was filtered and washed with methanol, then dried and stored for characterization. The final product was obtained as a powder with a yield of 52%. Found (%) by EA: C, 61.30; H, 4.501; N, 8.301.

### Analysis

Thermal studies (TGA–DSC) were performed using a Netzsch STA449 F3 Jupiter instrument. 5.0 mg of each sample was placed in α-alumina crucibles, with air serving as the purge gas at a flow rate of 50 mL min^−1^. The furnace temperature was ramped from 30 to 1000 °C at a constant heating rate of 10 °C min^−1^. Metal contents in the solid-state compounds were determined by complexometric titration with standard EDTA solution, following the conversion of the compounds to their oxides by ignition and subsequent dissolution in hydrochloric acid, as described by Caires et al. (2013). The melting point of the ligand was determined by differential scanning calorimetry (DSC) (Figure S1) using a Q20 instrument (TA Instruments), with air as the purge gas, a heating rate of 10 °C min^−1^, and a sample mass of 1.0 mg. Complementary measurements were performed using a digital melting point apparatus (Fisatom, model 431D), capable of analyzing up to three capillaries simultaneously over a 50–300 °C range. Carbon, hydrogen, and nitrogen contents were obtained through microanalysis using an EA 1110 CHNS-O Elemental Analyzer (CE Instruments). High-resolution mass spectrum (HRESIMS) with electrospray ionization was measured on an Agilent Q-TOF (6520 B model) spectrometer, operating in the positive mode. The sample was dissolved in methanol and infused into the ESI source at a flow rate of 3 mL min^–1^. FTIR/ATR analysis was performed using a Bruker Invenio R spectrometer, equipped with a diamond ATR unit, scanning the range of 4000–100 cm^−1^ with 32 scans at a resolution of 4 cm^−1^. Background spectra were automatically recorded and subtracted, and the resulting spectra were saved using OPUS software. Powder X-ray diffraction (PXRD) was employed to investigate the crystallinity and structural modifications induced by metal coordination. The measurements were performed using a Bruker D8 ADVANCE ECO diffractometer equipped with a Cu Kα radiation source (λ = 1.5406 Å), operating at 1000 W, within the 2θ range of 5–90°, using a step size of 0.025°, a counting time of 0.6 s per step, and a spinner speed of 15 rpm. The samples were dried, finely ground, and sieved through a 250-mesh screen to ensure homogeneity. The diffraction patterns were processed using OriginLab software with Gaussian peak fitting, and the average crystallite size (D) was estimated using the Scherrer equation (K = 0.9). UV–Vis spectra were recorded on a Bel Photonics UV-M51 spectrophotometer using quartz cuvettes with a 1.0 cm path length. 1Hand ^13^CNMR spectra were recorded on a Bruker Avance Neo spectrometer operating at 300 MHz for 1H and 75 MHz for 13C at 25 °C. DMSO-d₆ was used as the solvent, with tetramethylsilane (TMS) serving as the internal standard.

### CT-DNA binding experiments

#### UV–Vis titration

UV–Vis spectroscopy was used to study the binding of the ligand and Ni(II), Cu(II), and Zn(II) Schiff base complexes with calf thymus DNA (CT-DNA). DNA was dissolved in Tris-HCl buffer (pH 7.4), and its purity was confirmed by the A_260_/A_280_ ratio, which was found to be 1.8; this value indicates that there is no protein contamination. The DNA concentration was determined at 260 nm using an extinction coefficient of 6600 M^−1^ cm^−1^ (Jamshidvand et al. 2018). To ensure solubility and minimize interference, the compounds were prepared in 5% DMSO and 95% buffer, with a fixed concentration of 15 μM. DNA was then added stepwise from 0.0up to 193 μM, with equal amounts introduced into reference cuvettes for correction purposes. After gentle mixing and 2 min of equilibration, spectra were recorded, and absorbance changes were monitored. Binding constants (*K*_*b*_) were calculated from these variations using Eq. 1 (Wolfe et al. 1987), and all measurements were performed in triplicate to assess reproducibility.

1 $$\frac{{\left[ {\text{CT - DNA}} \right]}}{{\left( {\varepsilon {\mathrm{a}} - \varepsilon {\mathrm{f}}} \right)}} = \frac{{\left[ {\text{CT - DNA}} \right]}}{{\varepsilon {\mathrm{b}} - \varepsilon {\mathrm{f}} }} + \frac{1}{{{\mathrm{Kb}}\left( {\varepsilon {\mathrm{b}} - \varepsilon {\mathrm{f}}} \right)}}$$

where [CT-DNA] is the concentration of CT-DNA in base pairs, *ε*_a_ is the apparent extinction coefficient (calculated as absorbance divided by the compound's concentration), *ε*_f_ is the extinction coefficient of the free complex, and *ε*_b_ is the extinction coefficient of the complex in the fully DNA-bound state. The intrinsic binding constant *K*_*b*_ is obtained from the ratio of the slope to the intercept in the plot of [CT-DNA]/(*ε*_a_ − *ε*_f_) vs [CT-DNA]. The absorption band analyzed extended from 338 to 382 nm.

#### Study of complex–CT-DNA interaction by viscosity measurements

Viscosity measurements were performed using an Ostwald viscometer inserted in a water bath and maintained at 25 ± 0.5 °C.CT-DNA concentration was constant at 4.26 × 10^−3^ M, while the concentration of the synthesized compounds (1.00 × 10^−4^ M) was gradually increased to achieve different molar ratios of [complex]/[CT-DNA], ranging from 0 to 0.230. The flow time was measured using a stopwatch. Each sample was measured five times, and the average value was used for analysis. The effect of complex binding on DNA viscosity was examined by plotting (η/η_0_)^1/3^ against the ratio of [complex] to [DNA], where η and η_0_ represent the viscosities of CT-DNA solutions with and without the complex, respectively. Viscosity measurements were derived from the recorded flow times of DNA-containing solutions, corrected using the flow time of the buffer as a reference.

#### Ethidium bromide-displacement studies

The interaction of the synthesized compounds with CT-DNA was further investigated using an ethidium bromide (EB) displacement fluorescence assay. EB-bound CT-DNA solutions were prepared in Tris buffer (4.5 mM Tris-HCl, 0.50 mM Tris base, and 50 mM NaCl, pH 7.4). Fluorescence measurements were carried out using a fluorescence spectrophotometer. The initial assay solution in a 3.0 mL quartz cuvette contained CT-DNA (1.0 × 10^−4^ M) and EB (2.0 × 10^−5^ M). Emission spectra were recorded in the range of 500–800 nm with an excitation wavelength of 280 nm. The EB–CT-DNA system exhibited a maximum emission band at approximately 600 nm. Stock solutions of the synthesized compounds (1.0 × 10^−4^ M) were prepared in DMSO and added stepwise to the EB–CT-DNA solution in 15 μL aliquots. Ten successive additions were performed, corresponding to a total added volume of 150 μL. The compound concentrations in the cuvette were calculated considering the increase in total assay volume after each addition, resulting in a final concentration range of 0–4.762 × 10^−6^ M.

The concentrations of CT-DNA and EB were maintained approximately constant throughout the experiment, while the concentration of the tested compound was progressively increased. The final DMSO content in the assay mixture was sufficiently low and did not interfere with the fluorescence measurements.

The gradual decrease in fluorescence intensity of the EB–CT-DNA system upon addition of the compounds was attributed to the displacement of EB from its binding sites on DNA by the tested compounds. The quenching data were analyzed using the Stern–Volmer equation (Eq. 2), and the Stern–Volmer quenching constants (Ksv) were determined from the slopes of the corresponding plots. The obtained Ksv values were subsequently used to evaluate the relative DNA-binding affinities of the synthesized compounds.

2 $${\mathrm{I}}_{0} /{\mathrm{I}} = {\mathrm{K}}_{{{\mathrm{sv}}}} \left[ {\mathrm{Q}} \right] + 1$$

### BSA-binding experiment

#### Fluorescence spectroscopy

The protein binding study was carried out using tryptophan fluorescence quenching experiments with bovine serum albumin (BSA, 1.5 μM) in Tris–HCl buffer (pH 7.4). The decrease in the emission intensity of BSA tryptophan residues at 350 nm was monitored upon excitation at 280 nm in the presence of the compounds, which were added stepwise in six successive concentrations up to 90 μM. Before measurement, the solutions were allowed to equilibrate for two minutes, and each experiment was carried out in triplicate to ensure reproducibility. The results were evaluated using the Stern–Volmer equation (Eq. 3) and a double-logarithmic plot of the fluorescence data (Eq. 4), as previously reported by Moreira et al. (2025) and Sharma and Pathak (2024).

3 $${\mathrm{F}}_{0} /{\mathrm{F}} = {1} + {\mathrm{K}}_{{{\mathrm{sv}}}} \left[ {\mathrm{Q}} \right] = {1} + {\mathrm{K}}_{{\mathrm{q}}} {\mathrm{t}}_{0} \left[ {\mathrm{Q}} \right]$$

4 $$\log \frac{{\left( {{\mathrm{F}}0 - {\mathrm{F}}} \right)}}{{\mathrm{F}}} = \log {\mathrm{K}}_{{\mathrm{b}}} + {\mathrm{nlog}}\left[ {\mathrm{Q}} \right]$$

where F_0_ and F are the fluorescence intensities in the absence and presence of the quencher, respectively, and [Q] is the quencher concentration. K_sv_ is the Stern–Volmer quenching constant, which is related to the bimolecular quenching rate constant (K_q_) by the equation K_sv_ = K_q_τ_0_, where K_q_ is the bimolecular quenching rate constant, and τ_0_ is the average fluorescence lifetime (τ_0_ = 10^−8^ s) of the fluorophore in the absence of the quencher. Equation (3) was used to determine K_sv_ from the linear regression plot of F_0_/F versus [Q]. The binding constant (K_b_) and the number of binding sites (n) for the interaction between the compounds and BSA were determined by constructing a double logarithmic plot of the fluorescence data using Eq. 4.

### Molecular docking

The three-dimensional structures of the ligand and metal complexes were constructed using the Avogadro software and optimized using the semi-empirical PM7 method implemented in MOPAC 2016 (Stewart 2016). The optimized geometries were used for molecular docking calculations. Ligand preparation was performed in Avogadro by adding polar hydrogen atoms and assigning Gasteiger charges (Sanner 1999). The crystal structure of double-stranded B-DNA (PDB ID: 4U8A, resolution 1.48 Å) was retrieved from the RCSB Protein Data Bank. The receptor was prepared using UCSF Chimera (version 1.17.3) by removing crystallographic water molecules, adding polar hydrogen atoms, and assigning Kollman charges. Protonation states were assigned to represent physiological conditions (pH 7.4), with the phosphate groups maintained in their deprotonated, negatively charged form. The co-crystallized ligand was retained for subsequent docking protocol validation (Mukherjee et al. 2010).

The optimized structures were converted to PDBQT format, and the metal complexes were treated as intact coordination entities, preserving their coordination geometries throughout the docking procedure. A blind docking approach was performed using AutoDock Vina (implemented in PyRx), which utilizes an iterated local search global optimizer (BFGS gradient-based local search). Although Gasteiger and Kollman's partial charges were assigned during the preparation steps to maintain standard protocol compatibility, it is worth noting that AutoDock Vina’s empirical scoring function omits explicit electrostatic charge calculations, as these interactions are implicitly accounted for within its parameterized hydrophobic and hydrogen-bonding terms. The grid box was centered at X = 10.135, Y = 1.979, and Z = 23.036, with dimensions of 25.547 × 29.989 × 45.539 Å and a standard grid spacing of 1.0 Å. The search exhaustiveness was set to 12.

The molecular docking protocol was validated by redocking the co-crystallized ligand present in the 4U8A structure. The lowest-energy docking pose was compared with the experimental crystallographic conformation, yielding a heavy-atom RMSD of 0.593 Å. As this value is well below the commonly accepted threshold of 2.0 Å, the docking protocol was considered reliable. The excellent overlap between the crystallographic and redocked conformations is presented in Figure S3b (Supplementary Material) and provides additional support for the docking results discussed in “Molecular docking” section.

## Results and discussion

### Thermal study (TGA–DSC)

**Fig. 1:**
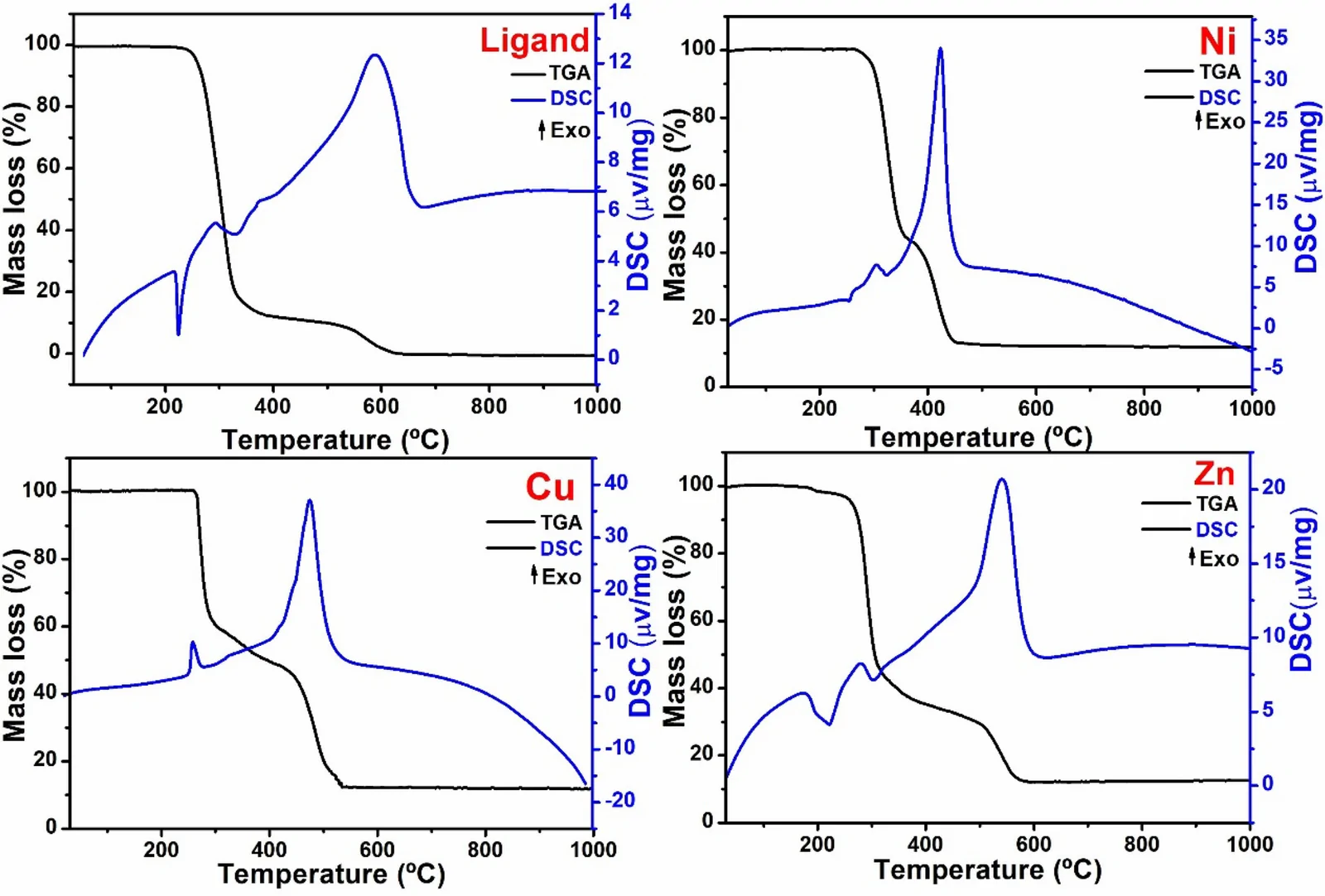
TGA–DSC curves of ligand(L), Ni(II), Cu(II), and Zn(II) complexes, endothermic (↓) and exothermic (↑) events. (air, 30–1000 °C, 10 °C/min, 5 mg sample mass)

The TGA–DSC profiles of the ligand (L) and its Ni(II), Cu(II), and Zn(II) complexes (Fig. 1) reveal distinct thermal behaviors, all characterized by relatively high thermal stability: 193 °C (L), 240 °C (Ni), 257 °C (Cu), and 174 °C (Zn). The free ligand melts at 205.6 °C and decomposes between 234 and 400 °C, as indicated by a weak exothermic peak at 274 °C in the DSC curve. This decomposition leads to the formation of a relatively stable carbonaceous residue derived from the conjugated aromatic structure, observed in the TGA curve as a plateau between 400 and 636 °C. The complete oxidative degradation of this residue occurs through an intense exothermic event at 570 °C, leaving no residue above 636 °C.

For the metal complexes, except for the zinc compound, no mass losses were observed at low temperatures, corroborating the absence of solvent or coordinated water molecules in their polycrystalline structures. This initial stability is characteristic of structurally well-defined complexes and suggests strong metal–ligand interactions within the bidentate coordination environment of the Schiff base(Ejidike et al. 2025, 2021).

In the Ni(II) complex, a small endothermic signal at 253 °C (DSC) is attributed to melting without significant mass loss. Thermal decomposition begins above 264 °C and proceeds in two main steps: the first up to 357 °C, with a mass loss of 55.33%, and the second up to 600 °C, with an additional loss of 32.49%, resulting in a stable NiO residue corresponding to 12.18%. These results are consistent with a metal-to-ligand stoichiometric ratio of 1:2, suggesting the formula [Ni(L)₂].

For the Cu(II) complex, the decomposition occurs in three steps: the first, between 257 and 289 °C, with a mass loss of 36.87%, accompanied by a weak exothermic peak at 268 °C; the second, between 289 and 437 °C, with a loss of 16.8%, associated with the gradual degradation of the ligand and the formation of a carbonaceous intermediate; and the third, between 437 and 542 °C, with a loss of 33.88%, attributed to the oxidation/combustion of this carbonaceous residue, evidenced by an intense exothermic peak at 485 °C. At the end of the process, a stable CuO residue corresponding to 12.45% remains. The total organic mass loss (87.55%) is in agreement with a ligand-to-metal ratio of 2:1, confirming the proposed composition [Cu(L)₂].

The Zn(II) complex remains stable up to 174 °C, followed by a slight mass loss of approximately 2% between 174 and 208 °C, associated with an asymmetric endothermic peak with a shoulder in the DSC curve, suggesting a melting process accompanied by slight decomposition. According to studies reported in the literature (Sánchez-Férez et al. 2021; Shiu et al. 2002), depending on the structural arrangement and crystal packing, solvent molecules may remain occluded within the crystal lattice, i.e., physically trapped through weak intermolecular interactions such as hydrogen bonding, van der Waals forces, or confinement within cavities/channels of the crystalline network. This behavior may be suggested for the Zn(II) complex in the present study. After this small initial event, decomposition occurs in three steps: (i) 251–304 °C, with a mass loss of 48.17% and an exothermic peak at 278 °C; (ii) 304–502 °C, with a loss of 20.79%, attributed to the formation of a carbonaceous intermediate; and (iii) 502–600 °C, with a loss of 16.84%, related to the oxidation/combustion of the carbonaceous material, producing an intense exothermic peak at 538 °C and resulting in a stable ZnO residue corresponding to 12.20%. Based on these results, the proposed stoichiometry for the complex is [Zn(L)₂]·0.5CH₃OH, indicating the presence of half a mole of occluded methanol.

Regarding the thermal decomposition pattern, it is possible to suggest, based on the main bond energies involved in the ligand structure (N–N ≈ 160–180 kJ mol^−1^; C–C ≈ 340–370 kJ mol^−1^; C–O ≈ 340–370 kJ mol^−1^), some structurally more susceptible points for thermal cleavage. The rupture of these bonds may initially lead to the release of small amounts of higher molecular weight volatile organic fragments. However, under an air atmosphere, the intense oxidation of the organic matter predominantly produces CO₂ and CO as final volatile products. Thus, some plausible intermediate fragments may include species such as 2-MeO–C₆H₄–CH=CH–CH=N–, 2-MeO–C₆H₄–CHO, 2-MeO–C₆H₄–, C=C–C=N–, 2-MeO–C₆H₄(F)–, F–C₆H₄–, and F–C₆H₄–CHO. Nevertheless, the specific identification of these fragments requires coupled thermoanalytical techniques such as TGA-FTIR or TGA-MS.

Finally, to validate the stoichiometric ratios inferred from the TGA–DSC data, elemental analyses (EA) were performed to determine the percentages of C, H, and N, while complexometric titration with EDTA was used to quantify the metal content expressed in the form of its respective oxides. The experimental values obtained by TGA, EA, and EDTA titration showed good agreement with the calculated theoretical values, consistent with a 1:2 metal-to-ligand stoichiometric ratio, as presented in Table 1. These analytical results are further strongly supported by the mass spectrometry data (see Supplementary Material, Figure S2).

**Table 1:**
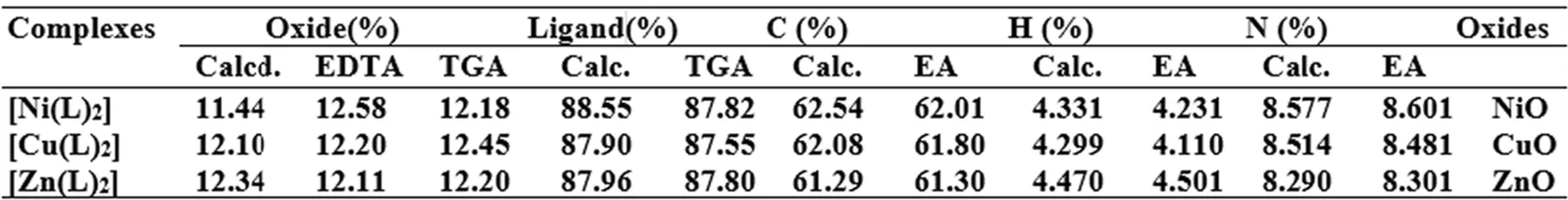
Calculated and experimental values for oxide content, ligand fraction, and elemental composition (C, H, and N) of the [M(L)₂] complexes (M = Ni, Cu, and Zn), as determined by TGA, elemental analysis (EA), and EDTA titration

Oxide (%) = theoretical and experimental metal oxide contents obtained from TGA residue (> 650 °C) and EDTA titration (expressed as metal oxide); Ligand (%) = theoretical and experimental organic contents obtained from total TGA mass loss; Calcd. = calculated values; EA = elemental analysis; C (%), H (%), and N (%) = calculated and experimental elemental compositions

### FTIR/ATR spectroscopic analysis

**Fig. 2:**
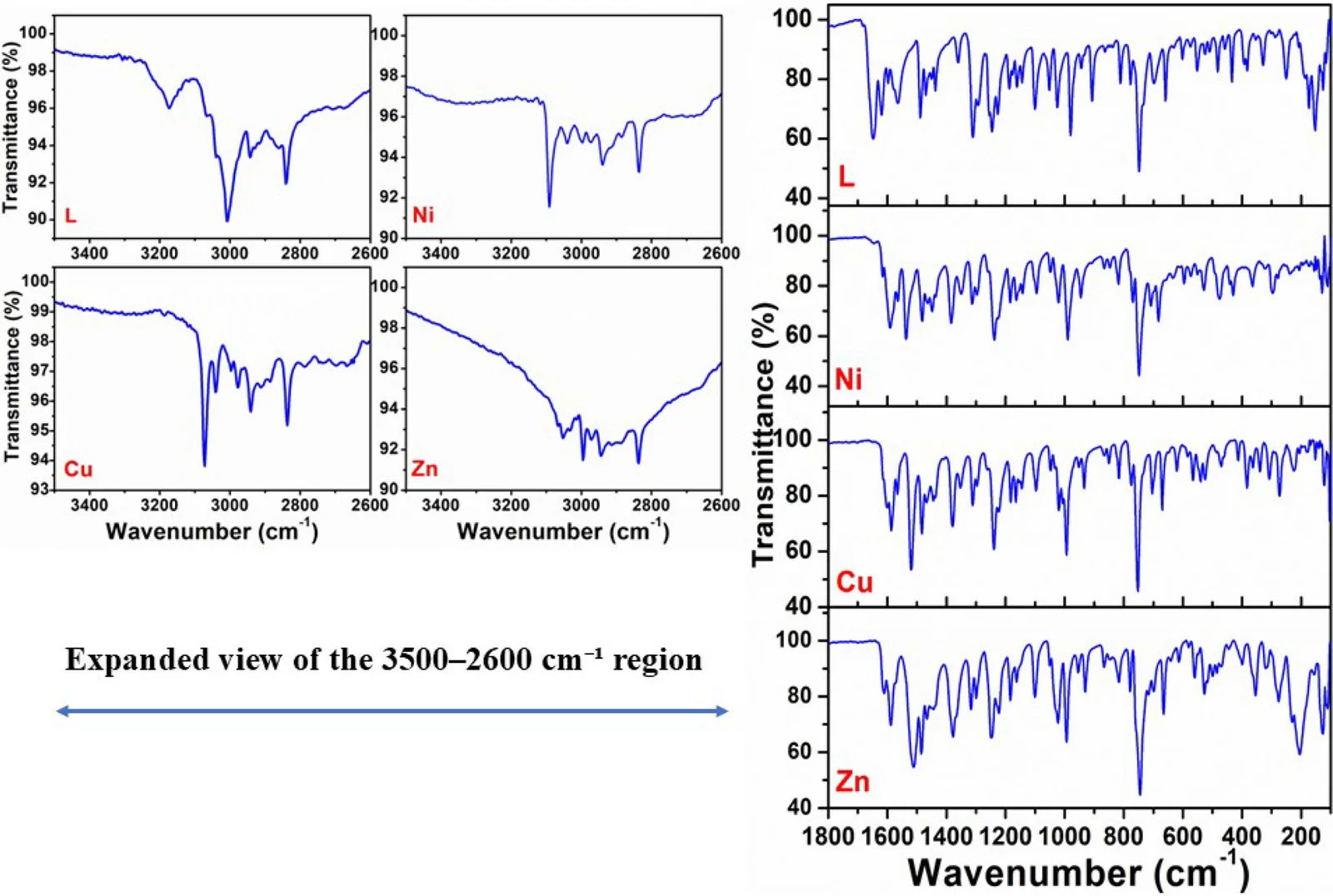
FTIR/ATR spectra of the ligand (L) and Ni(II), Cu(II), and Zn(II) complexes, recorded in the range of 4000–100 cm^−1^, 32 scans at a resolution of 4 cm^−1^

The FTIR/ATR analysis shown in Fig. 2 presents the spectra of the organic ligand and its Ni(II), Cu(II), and Zn(II) metal complexes. The spectrum of the free ligand exhibits a broad and well-defined band at 3172 cm^−1^, attributed to the ν(N–H) stretching vibration of the hydrazone group (–NH–N=), characteristic of hydrogen bonding to nitrogen. The absorption bands at 3008, 2943, and 2840 cm^−1^ correspond to the ν(C–H) stretching vibrations of aromatic and aliphatic (methoxy) groups, respectively. A strong band observed at 1647 cm^−1^ is assigned to the ν(C=O) stretching vibration of the carbonyl group conjugated with the aromatic system, while the band at 1566 cm^−1^ is associated with the ν(C=N) stretching vibration of the azomethine (hydrazone) moiety. The band at 1489 cm^−1^ is attributed to ν(C=C) vibrations of the aromatic rings. Intense absorptions in the 1309–1101 cm^−1^ region correspond to ν(C–O–C) stretching modes of the methoxy group, whereas the intense band at 748 cm^−1^ is assigned to out-of-plane =C–H bending vibrations (Awolope et al. 2022; Dikio et al. 2017; Ejidike et al. 2025; Moreira et al. 2022; Silverstein et al. 2005).

After complexation, the ν(C=O) band shifts from 1647 cm^−1^ to 1591 cm^−1^ (Ni), 1587 cm^−1^ (Cu), and 1589 cm^−1^ (Zn), suggesting coordination of the carbonyl oxygen atom to the metal center. Similarly, the ν(C=N) band shifts from 1566 cm^−1^ to 1537 cm^−1^ (Ni), 1519 cm^−1^ (Cu), and 1510 cm^−1^ (Zn), which may be associated with the involvement of the imine nitrogen atom in metal–ligand coordination (Moreira et al. 2022; Nakamoto 2009). This interpretation is further supported by the disappearance of the ν(N–H) stretching band at 3172 cm^−1^ (see the NMR discussion section for additional support), which is present in the free ligand but absent in all metal complexes, indicating deprotonation of the hydrazone group upon coordination. For all spectra, the intense band in the 980–993 cm^−1^ region, observed at 980 cm^−1^ (ligand), 989 cm^−1^ (Ni), and 993 cm^−1^ (Cu and Zn), can be attributed to the asymmetric ν(C–O–C) stretching mode of the aromatic methoxy group (–OCH₃).

In the far-infrared region (400–100 cm^−1^), new bands appear that are absent in the spectrum of the free ligand, corresponding to ν(M–O) and ν(M–N) vibrations. These assignments are consistent with the characteristic vibrational frequencies reported for metal–oxygen and metal–nitrogen stretching modes in coordination compounds (Nakamoto 2009). The corresponding bands are located at 474, 430, and 297 cm^−1^ for Ni; 384, 274, and 226 cm^−1^ for Cu; and 526, 355, 276, and 204 cm^−1^ (broad and intense) for Zn(II). The broad absorption around 204 cm^−1^ for the Zn(II) complex may be attributed to combined ν(Zn–O,N) vibrations, which are typical of bidentate coordination modes (Moreira et al. 2022; Nakamoto 2009).

Taken together, the observed shifts in the ν(C=O) and ν(C=N) bands, together with the appearance of ν(M–O) and ν(M–N) vibrations, support the conclusion that the ligand acts as an N,O-bidentate chelating agent. These spectroscopic features are consistent with coordination through the oxygen atom of the carbonyl/enolate moiety and the azomethine nitrogen atom, leading to the formation of the characteristic five-membered chelate ring commonly observed for acylhydrazone metal complexes.

### ^1^H and ^13^CNMR analysis

**Fig. 3:**
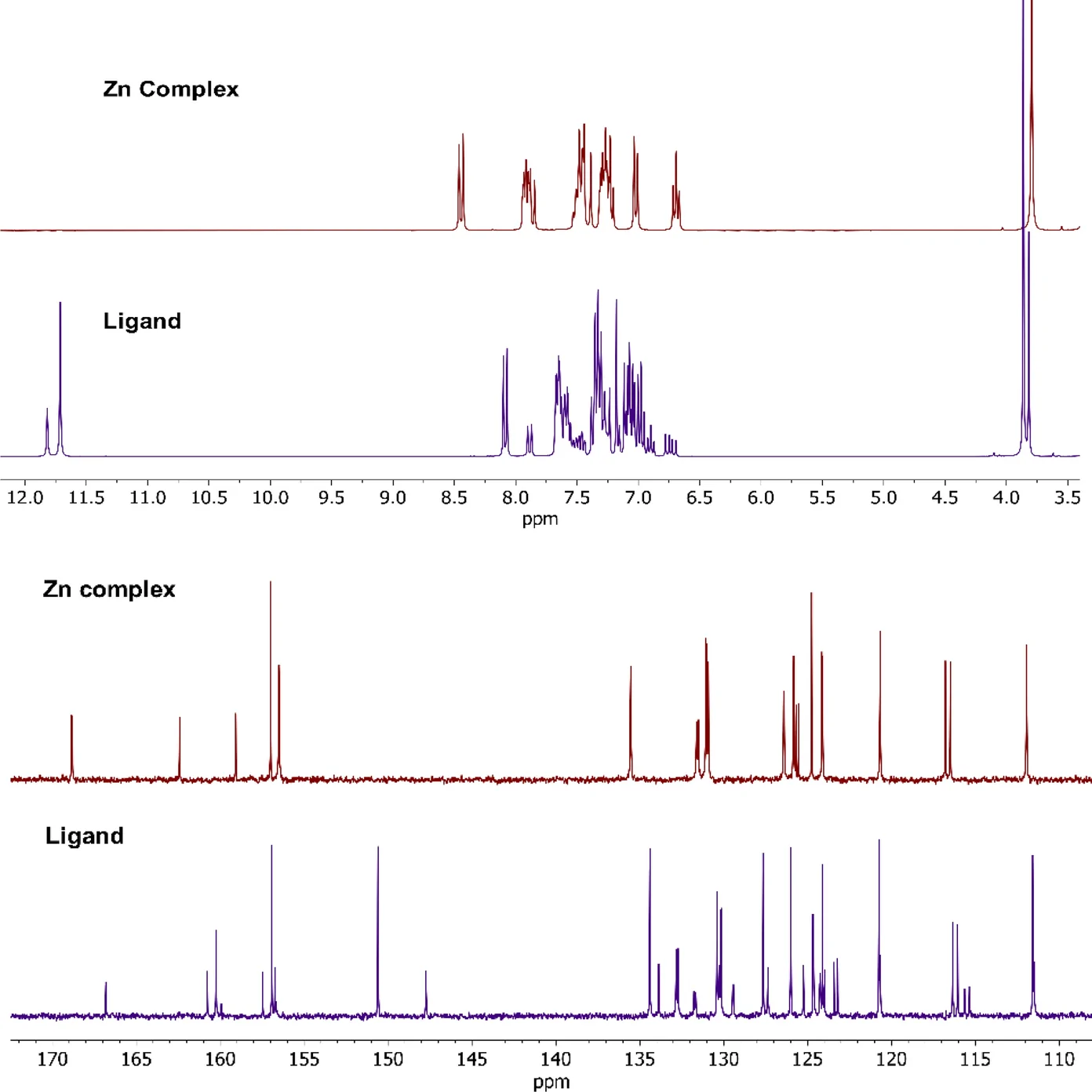
Comparison of the ^1^H and ^13^C NMR spectra of the free ligand and its zinc complex

Because the Zn(II) complex is diamagnetic, the proposed N,O-bidentate chelation through the carbonyl oxygen and the imine nitrogen is strongly supported by the marked change in the ^1^H NMR signal pattern upon coordination (Fig. 3). While the free ligand displays two sets of duplicated resonances arising from the coexistence of E-trans and E-cis conformers in solution, the zinc complex exhibits a single, well-resolved set of signals. This behavior is fully consistent with conformational locking of E-trans and E-cis conformers in solution, induced by N,O-chelation, which restricts rotation along the C–N amide bond and enforces a single trans geometry.

Coordination of the imine group is further evidenced by the downfield shift of the azomethine proton (Δδ ≈ 0.3 ppm), reflecting decreased electron density on the C=N moiety upon metal binding. Finally, the anionic nature of the coordinated ligand is confirmed by the disappearance of the broad NH resonance above 11 ppm, which is present in the neutral form of L but absent in the zinc complex.

The ^13^C NMR spectrum is consistent with the proposed ligand structure and corroborates the formation of the acylhydrazone framework. Although the coexistence of E-trans and E-cis conformers in solution, combined with long-range ^13^C–^1^⁹F couplings, leads to extensive signal splitting and overlap, the observed resonances are in agreement with the expected carbon environments. Therefore, while a complete individual assignment of all carbon signals is not straightforward, the spectrum provides strong support for the proposed molecular structure of the free ligand and the Zn(II) complex.

It should be noted that the present NMR discussion is restricted to the diamagnetic Zn(II) complex. Since Ni(II) and Cu(II) may exhibit paramagnetic behavior depending on their coordination geometry, ^1^H and ^13^C NMR spectroscopy does not provide comparable structural information for these complexes. Therefore, the proposed molecular compositions and coordination modes for the Ni(II) and Cu(II) complexes are based on the combined evidence obtained from FTIR, HRESIMS, elemental analysis, EDTA titration, PXRD, TGA–DSC, and comparison with related complexes reported in the literature. Although these complementary data consistently support the proposed molecular compositions and the N,O-bidentate coordination mode, magnetic susceptibility measurements would provide valuable complementary information for a more comprehensive structural characterization, particularly for the Ni(II) complex.

### Powder X-ray diffraction (PXRD)

**Fig. 4:**
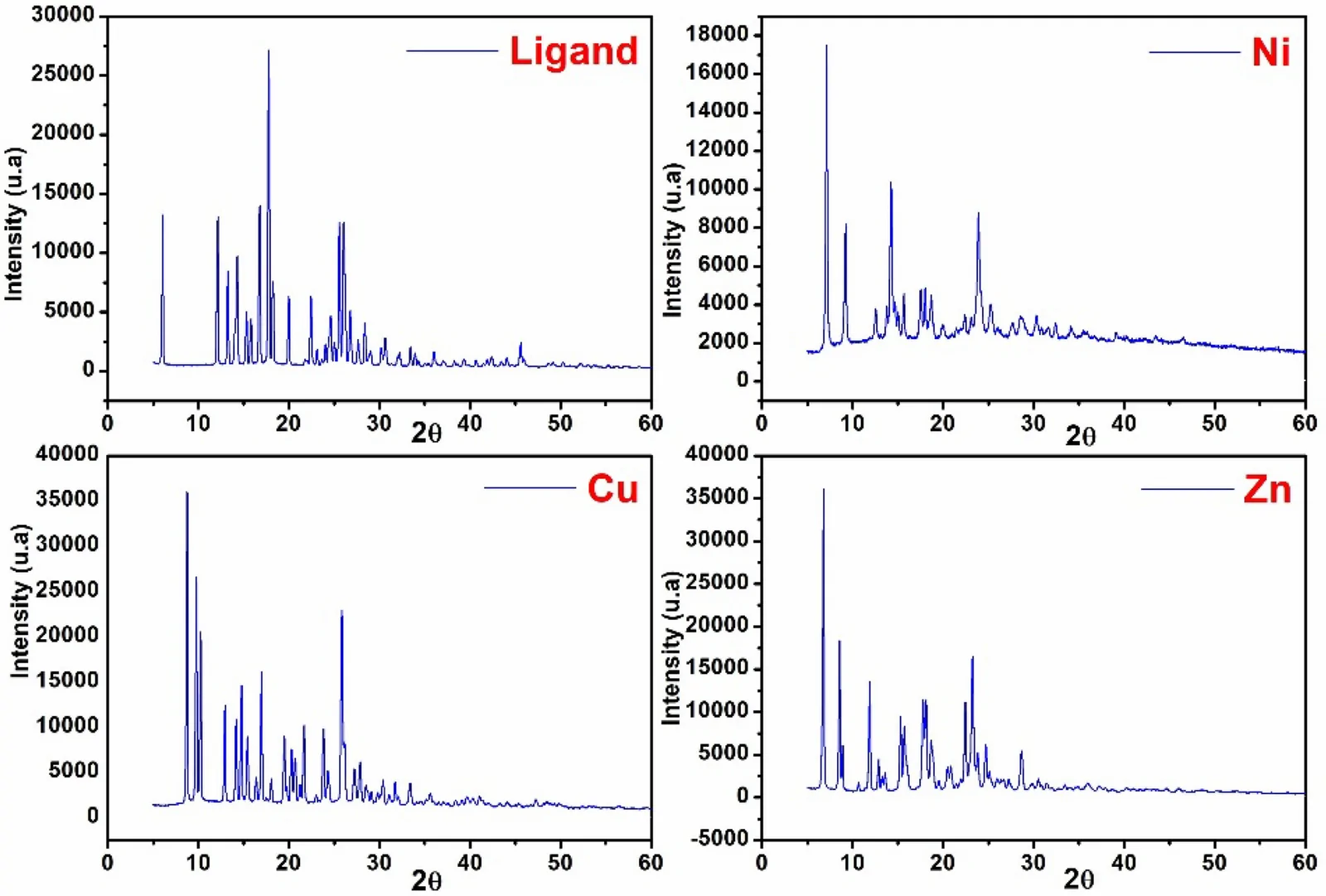
Powder X-ray diffraction (PXRD) of the ligand, Ni(II), Cu(II), and Zn(II) complexes

The Powder X-ray diffraction data are shown in Fig. 4. The free ligand exhibited sharp and intense diffraction peaks, indicating its highly crystalline nature. The Scherrer equation provided an average coherent crystallite size of 41 ± 4 nm, suggesting the presence of well-ordered crystalline domains. Upon metal coordination, the diffraction patterns of all complexes showed noticeable shifts in peak positions and variations in relative intensity, consistent with changes in crystal packing and coordination environment after complexation (Ejidike et al. 2025; Kamali and Amani 2024; Saif et al. 2016). The Ni(II) complex displayed broader reflections and a smaller average coherent crystallite size of 28 ± 8 nm, suggesting increased microstructural disorder and a reduction in coherent domain size. In contrast, the Cu(II) and Zn(II) complexes retained relatively sharp reflections, with average coherent crystallite sizes of 36.8 ± 6.7 nm and 38 ± 8 nm, respectively, indicating that these materials remain crystalline, although with moderate structural reorganization compared to the free ligand. The observed trend in coherent crystallite size was: ligand (41 ± 4 nm) > Cu(II) (37 ± 7 nm) ≈ Zn(II) (38 ± 8 nm) > Ni(II) (28 ± 8 nm), suggesting that metal coordination partially affects crystallinity, with the most pronounced effect observed for Ni(II).

Taken together, the PXRD patterns revealed changes in diffraction profile, peak broadening, and coherent crystallite-domain size after metal coordination, indicating modifications in the crystalline organization of the ligand upon complex formation. These results are consistent with the formation of structurally distinct polycrystalline coordination compounds and are in agreement with the FTIR and TGA–DSC analyses.

### UV–Vis study

**Fig. 5:**
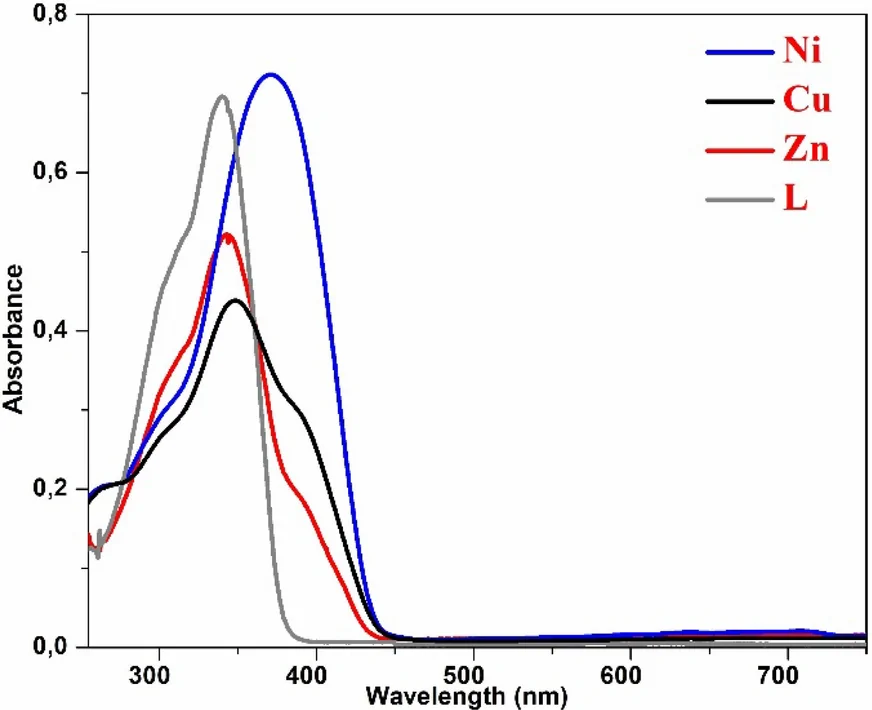
UV–Vis absorption spectra of the ligand (L) and its Ni(II), Cu(II), and Zn(II) complexes recorded in DMSO at a concentration of 20 μM

The UV–Vis absorption spectra of the ligand and its Ni(II), Cu(II), and Zn(II) complexes are shown in Fig. 5. The free ligand exhibited an intense absorption band centered in the 350–360 nm region, mainly attributed to intraligand π → π* and n → π* electronic transitions associated with the conjugated hydrazone framework. Upon metal coordination, significant spectral changes were observed for all complexes, including shifts in the absorption maxima, band broadening, and increased absorption extending toward the visible region, indicating perturbation of the ligand-centered electronic transitions and possible ligand-to-metal charge transfer contributions(Moreira et al. 2022).

Among the complexes, the Ni(II) derivative exhibited the most pronounced bathochromic shift and spectral broadening, suggesting stronger metal–ligand electronic interaction and greater perturbation of the conjugated system compared to the Cu(II) and Zn(II) complexes. The Cu(II) complex also displayed broadened absorption bands, consistent with electronic redistribution after coordination, although no clearly resolved d–d transitions could be distinguished from the intense ligand-centered and charge-transfer absorption envelope. For the Zn(II) complex, the spectrum remained predominantly governed by ligand-centered transitions, as expected for a d^1^⁰ metal ion, with the observed spectral shifts supporting coordination-induced modification of the ligand electronic environment.

The observed spectral changes support the formation of metal complexes with distinct electronic environments. However, since no well-resolved d–d transitions were observed under the experimental conditions, the UV–Vis data alone do not allow an unambiguous assignment of the coordination geometry. Therefore, the data were interpreted cautiously and correlated with the FTIR, PXRD, and TGA–DSC results, which together provide complementary evidence for successful metal–ligand coordination and the formation of structurally distinct complexes (Ejidike and Ajibade 2015).

### DNA binding study

#### Absorption spectroscopic studies

UV–visible spectroscopy has long been a trusted method for exploring how small molecules, including various potential drugs, interact with DNA. In this experiment, the compound was kept at a constant concentration while increasing amounts of DNA were added. As the DNA binds, the compound’s absorption spectrum typically changes, with peaks either increasing or decreasing in intensity; these changes are a clear sign that binding is taking place between the compound and the DNA (Channar et al. 2019).

**Fig. 6:**
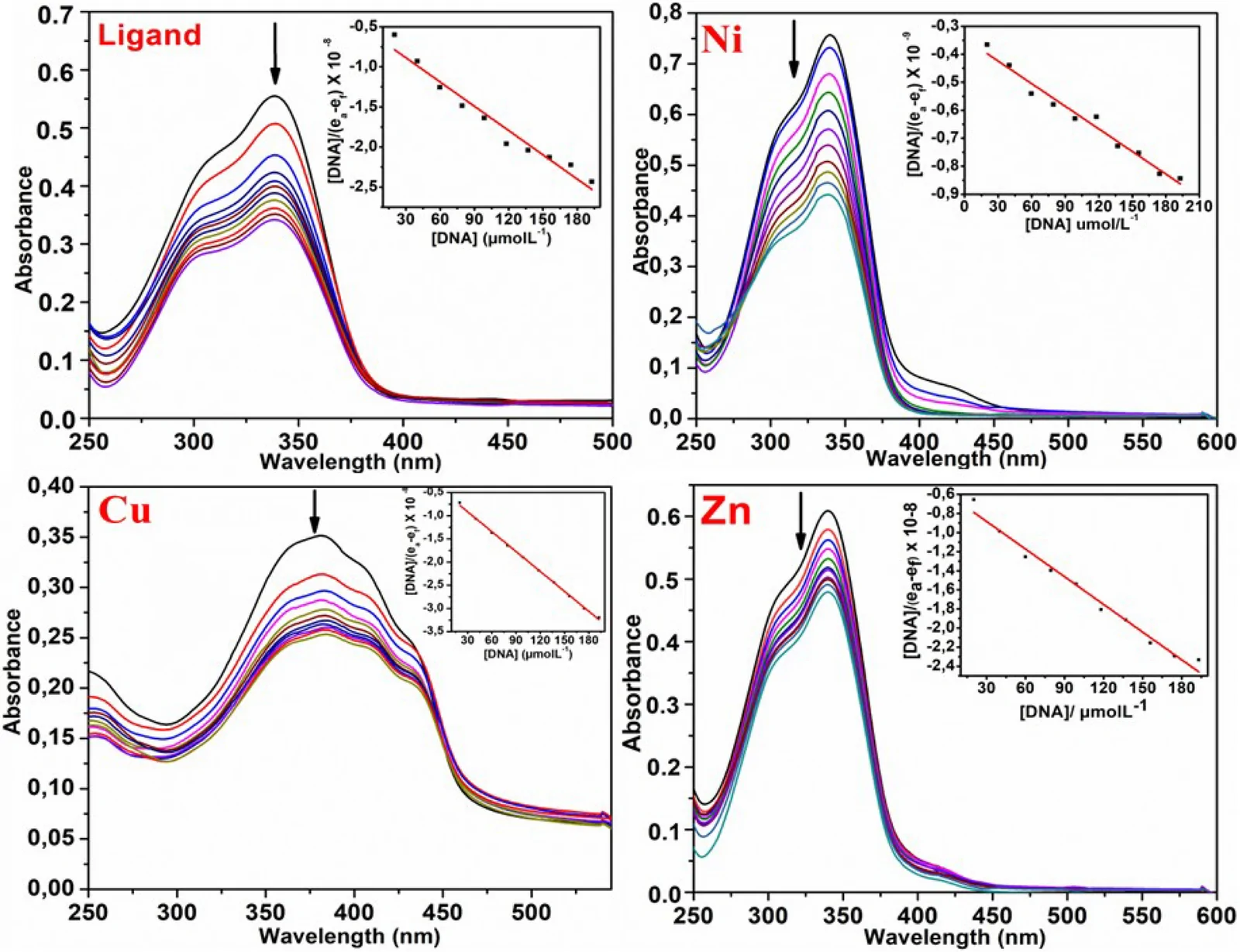
UV–Vis absorption spectra of the ligand (L), Ni(II), Cu(II), and Zn(II) complexes in the presence of increasing concentrations of CT-DNA. Arrows indicate changes in absorbance upon DNA addition. Insets: plots of [DNA] vs [DNA]/(εa-εf). The absorption band was centered around 338–382 nm

Upon the stepwise addition of CT-DNA to the ligand and its complexes, all compounds exhibited a moderate decrease in absorption intensity (hypochromism), with reductions ranging from 20.55 to 41.02%, and no accompanying red shift in wavelength was observed (Fig. 6, Table 2). This decrease in absorbance is commonly attributed to π–π stacking interactions between the aromatic parts of the compounds and the base pairs of DNA. This kind of spectral change is generally taken as a sign that the compounds interact with DNA either through partial intercalation or by fitting into the grooves of the double helix(Mushtaq and Naseer 2025).

**Table 2:** CT-DNA binding constants (*K*_*b*_) for the compounds

Compound	λ (nm)	*K*_*b*_(Lmol^−1^)	Hypochromism (%)
Ni	341	(5.07 ± 0.89) × 10^4^	41.02
Cu	382	(3.59 ± 0.55) × 10^4^	28.98
Zn	341	(1.70 ± 0.32) × 10^4^	20.55
L	340	(2.91 ± 0.46) × 10^4^	35.53

The binding constants (*K*_*b*_), obtained using the Benesi-Hildebrand method (Eq. 1), were found to be in the order of 10^4^ L mol^−1^ (Table 2). These values are lower than those reported for classical intercalators such as ethidium bromide (*K*_*b*_ ≈ 10⁶ L mol^−1^), which points to a non-classical binding mode(Strothkamp and Strothkamp 1994; Suh and Chaires 1995). The obtained *K*_b_ values are comparable to those already reported for some groove-binding Schiff base agents, such as [Cu_2_L_2_(NO_3_)_2_]·2CH_3_OH, 1.3 ± 0.2 × 10^4^ L mol^−1^] (Goudarziafshar et al. 2022), [Co (bpy)_2_(CNOIP)]^3+^, *K*_*b*_ = 5 × 10^4^ M^−1^] (Zhang et al. 2001), and a complex (1.67 × 10^4^ M^−1^) (Arjmand and Muddassir 2010). Similar values have also been reported for known groove binders, such as isatin (7.32 × 10^4^ M^−1^) (Shahabadi and Heidari 2014) and [Pt(Met)(DMSO)Cl]Cl (1.5 × 10^4^ M^−1^) (Kashanian et al. 2010).

Taken together, these results suggest that the synthesized ligand and its metal complexes interact with CT-DNA predominantly through groove binding. The observed binding affinity follows the order: Ni(II) complex > Cu(II) complex > free ligand > Zn(II) complex. This trend indicates that metal coordination influences the DNA-binding ability of the ligand, although the exact structural factors governing this behavior cannot be unequivocally established from the present data. The higher affinity observed for the Ni(II) and Cu(II) complexes may be associated with coordination-induced changes in the electronic distribution and molecular organization of the ligand framework, which can favor non-covalent interactions with DNA. In contrast, the Zn(II) complex exhibited the lowest affinity among the complexes, suggesting that the nature of the coordinated metal ion plays an important role in modulating the interaction with CT-DNA (Mahmoud et al. 2026).

Comparison of the free ligand and its metal complexes shows that complexation generally enhances CT-DNA interaction. Metal coordination can modify the electronic properties of the ligand and influence its interaction with the negatively charged DNA framework through a combination of hydrophobic, electrostatic, and van der Waals interactions. Consequently, the Ni(II) and Cu(II) complexes exhibited higher binding affinities than the free ligand, indicating that coordination contributes positively to DNA binding. These findings are consistent with the experimental results obtained from the spectroscopic and viscosity studies, which support the formation of stable DNA–compound adducts through a groove-binding mode.

The combined spectroscopic results suggest that these compounds likely bind to DNA through groove binding, as evidenced by the observed moderate hypochromism and relatively lower K_b_ values. This interpretation is further supported by viscosity measurements and fluorescence quenching studies (Table 3).

**Table 3:** Binding parameters for the interaction between BSA and metal complexes

Compound	*K*_*sv*_(10^5^ M^−1^)	*K*_*q*_(10^13^ M^−1^ s^−1^)	*K*_*b*_(M^−1^)	n
Ni	2.52	2.52	5.18 × 10^4^	1.04
Cu	4.12	4.12	5.80 × 10^5^	0.87
Zn	1.65	1.65	3.47 × 10^4^	1.04
L	1.11	1.11	1.37 × 10^4^	1.04

#### Viscosity titration method

Measuring changes in the viscosity of a DNA solution upon adding different concentrations of a compound is a reliable way to confirm DNA binding and to gain insight into the mode of interaction (Janjua et al. 2011). When a compound inserts itself between DNA base pairs, the relative viscosity (η/η^0^)^1/3^rises steadily with increasing compound-to-DNA ratio, reflecting the DNA helix becoming longer (Shahabadi et al. 2014). If the viscosity increases at first but then levels off or shows minimal variations at higher compound concentrations, it indicates a mixed binding mode (Channar et al. 2019). In contrast, a drop in viscosity suggests DNA denaturation, because the separated bases lead to a shorter, more flexible structure with lower resistance to flow(Janjua et al. 2011).

**Fig. 7:**
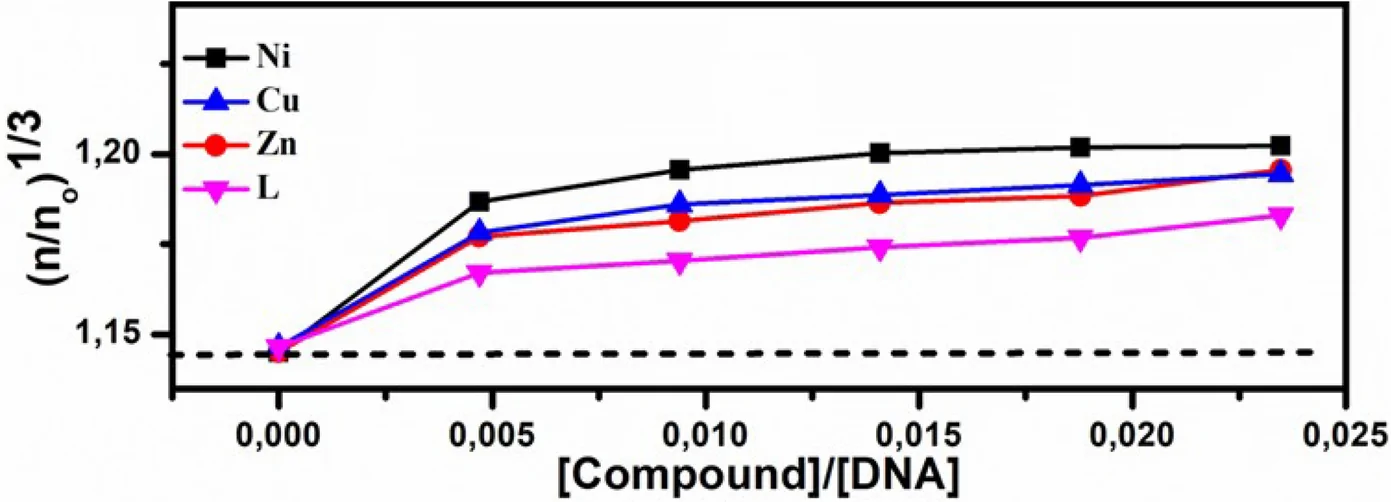
Effect of increasing concentrations of ligand(L),Ni(II), Cu(II) and Zn(II) complexes on the relative viscosity of CT-DNA at 25.0 ± 0.5 °C. [CT-DNA] = 4053 µM^−1^, pH 7.4, [compounds] = 100 µM

Figure 7 shows the plot of (η/η^0^)^1/3^ versus the [complex]/[DNA] molar ratio, highlighting how increasing concentrations of the synthesized compounds affect DNA viscosity. As observed, raising the [complex]/[DNA] ratio for the Ni(II), Cu(II), and Zn(II) complexes caused a gradual increase in relative viscosity. This suggests that the complexes interact with the DNA helix through a mixed binding mode, involving predominantly groove binding with a minor intercalative contribution (Eichhorn and Shin 1968; Senthilkumar et al. 2022).

Among the tested compounds, the Ni(II) complex caused the largest increase in DNA viscosity, followed by the Cu(II) and Zn(II) complexes, and finally the free ligand. This trend may be attributed to the stronger binding affinity of the Ni(II) and Cu(II) complexes, as evidenced by their higher K_b_ values, which are consistent with more effective interactions with the DNA helix. In contrast, the Zn(II) complex and the free ligand interact more weakly, resulting in smaller viscosity changes.

#### Ethidium bromide displacement studies

A fluorescence quenching study using ethidium bromide (EB) was carried out to explore the DNA binding mode of the synthesized ligand and metal complexes of Ni(II), Cu(II), and Zn(II). EB is a planar, cationic dye that exhibits strong fluorescence when intercalated between DNA base pairs(Köse et al. 2022; Lepecq and Paoletti 1967). Molecules that interact with DNA in a manner similar to EB can compete for the same binding sites, thereby reducing the fluorescence of the EB-DNA complex (Kakoulidou et al. 2025). In this study, a fixed EB–CT-DNA system was titrated with increasing concentrations of the ligand and metal complexes. All measurements were carried out under constant total volume conditions. The final concentrations of EB, CT-DNA, and the tested compounds were used instead of added volumes to ensure reproducibility and accurate comparison with other studies. The compounds were added from stock solutions, while maintaining a constant and minimal DMSO content to avoid solvent interference.

**Fig. 8:**
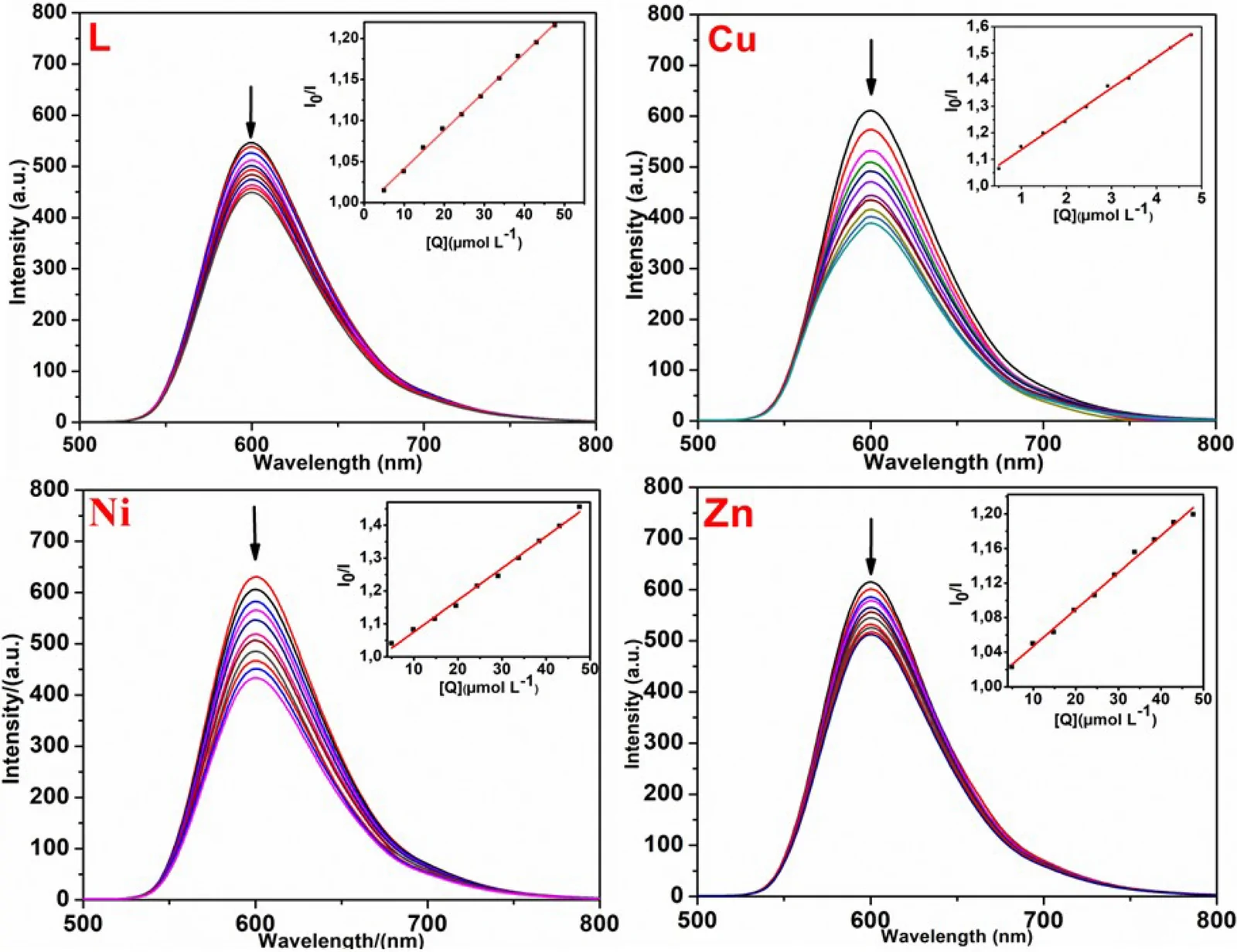
Fluorescence emission spectra of the EB–CT-DNA system before and after successive additions of the ligand (L), Ni(II), Cu(II), and Zn(II) complexes. The most intense spectrum corresponds to the EB–CT-DNA adduct in the absence of compound, while the remaining spectra were obtained after ten consecutive additions of ligand or complex. The gradual reduction in fluorescence intensity reflects the displacement of intercalated EB molecules from DNA by the tested compounds. Arrows indicate the direction of decreasing fluorescence intensity. Insets show the Stern–Volmer plots (I_0_/I vs. [Q]). Excitation wavelength: 280 nm; emission spectra recorded from 500 to 800 nm

After the addition of the synthesized ligand and its metal complexes to the EB-DNA system, a moderate decrease in fluorescence intensity (16.62–31.30%) was observed with increasing compound concentration. The observed decrease in fluorescence suggests that the compounds are competing with EB for DNA binding sites, effectively displacing EB from the DNA (Mohamed Subarkhan et al. 2016). As shown in Fig. 8, all compounds induced progressive quenching of EB–DNA fluorescence. The quenching constants (K_sv_) were found to follow the order: Cu(II) > Ni(II) > ligand > Zn(II), with values of 1.16 × 10^5^ M^−1^, 9.70 × 10^4^ M^−1^, 4.70 × 10^4^ M^−1^, and 4.26 × 10^4^ M^−1^, respectively. The higher quenching ability of the Cu(II) complex suggests a stronger ability to perturb the EB–DNA system, which may be attributed to its higher planarity and stronger π–π interaction capability, enabling more effective competition with EB at intercalative sites. In contrast, the Ni(II) complex, although showing stronger binding in UV–Vis studies, may preferentially interact through groove binding, resulting in slightly lower EB displacement efficiency compared to the Cu(II) complex. Fluorescence measurements were recorded at excitation and emission wavelengths appropriate for the EB–DNA system. Precautions were taken to minimize inner-filter effects caused by spectral overlap between EB, DNA, and the tested compounds (El‐Beltagy et al., 2025; Roy et al. 2025; Seleem et al. 2025; Tekerek et al. 2026).

Furthermore, the combination of the moderate hypochromism (< 50%) in fluorescence quenching and the magnitude of the K_sv_ values suggests that these compounds predominantly interact with DNA through a groove-binding mode rather than classical intercalation (Albaqami et al. 2023; Köse et al. 2022; Sherwani et al. 2022).

Collectively, the combined results from UV–Vis absorption, viscosity measurements, and ethidium bromide displacement experiments provide strong evidence that the synthesized compounds interact with CT-DNA, primarily through a groove-binding mode.

### BSA-binding experiment

#### Fluorescence spectroscopy

Serum albumin is an important blood protein and acts as a carrier for various molecular materials, including drugs. Serum albumin can serve as a macromolecular carrier for targeted delivery of anticancer agents, and its binding affinity with bioactive compounds is critical for drug bioavailability, distribution, and toxicity (Lintnerová et al. 2025). Bovine serum albumin (BSA) is widely used as a model protein due to its high structural similarity to human serum albumin (HSA)(De Souza et al. 2025).

**Fig. 9:**
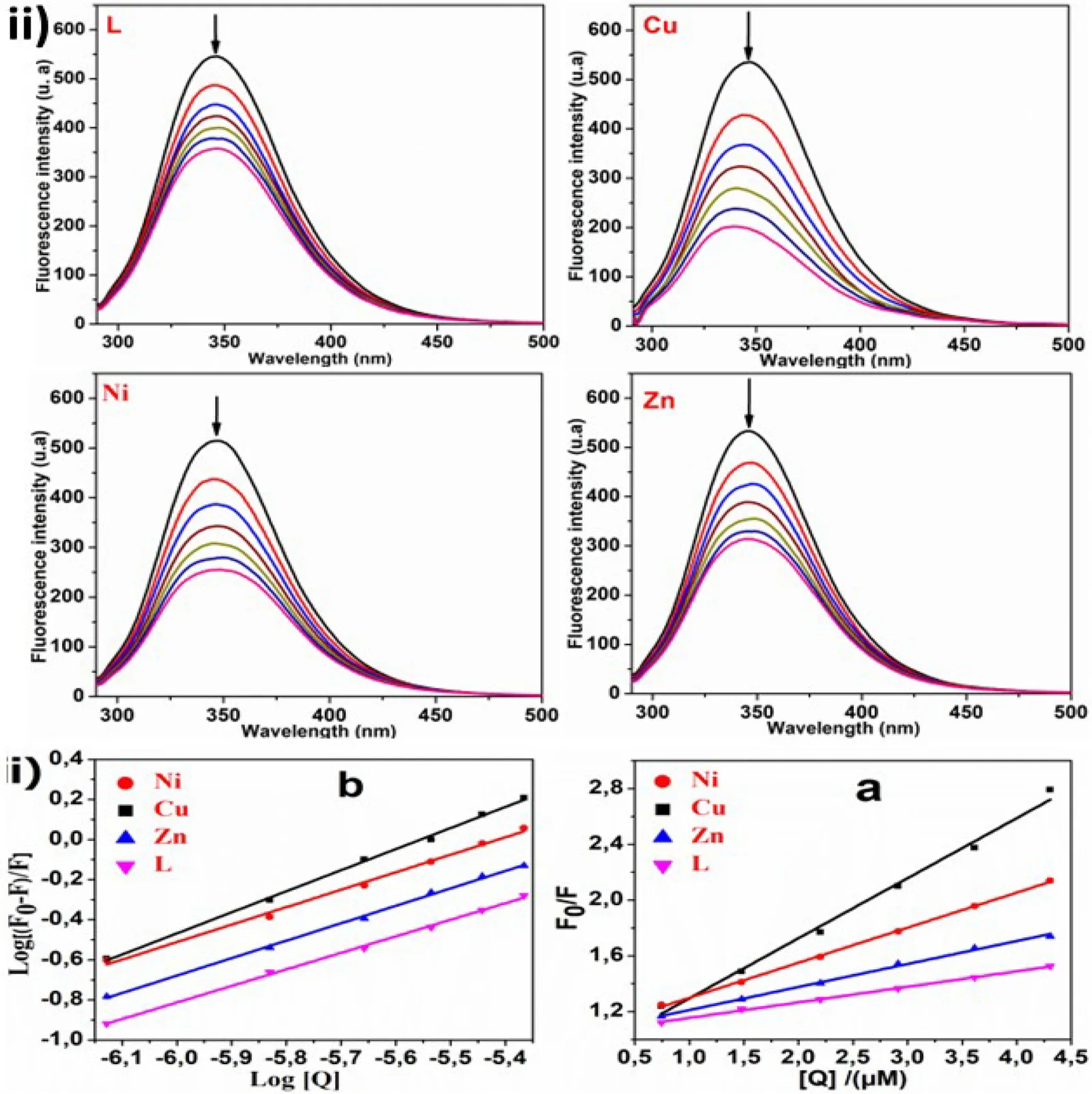
(**i**) Suppression of BSA fluorescence upon addition of different concentrations of the ligand (L), Ni(II), Cu(II), and Zn(II) complexes. (**ii**) (*a*) Stern–Volmer plots and (*b*) double-logarithmic plots obtained from the fluorescence titration of BSA with the compounds. The samples were excited at 280 nm, and the emission intensity was monitored at 350 nm

The fluorescence emission of BSA, primarily arising from Trp134 and Trp212, was observed at 350 nm upon excitation at 280 nm (Fig. 9i). The synthesized compounds, including the ligand and its Ni(II), Cu(II), and Zn(II) complexes, caused significant quenching of BSA fluorescence, indicating increased hydrophobicity and decreased polarity in the microenvironment surrounding the tryptophan residues (Lintnerová et al. 2025). The quenching analysis points to a predominantly static component, as the calculated k_q_ values (k_q_ ~ 1013 M^−1^ s^−1^) exceed the typical.

**Fig. 10:**
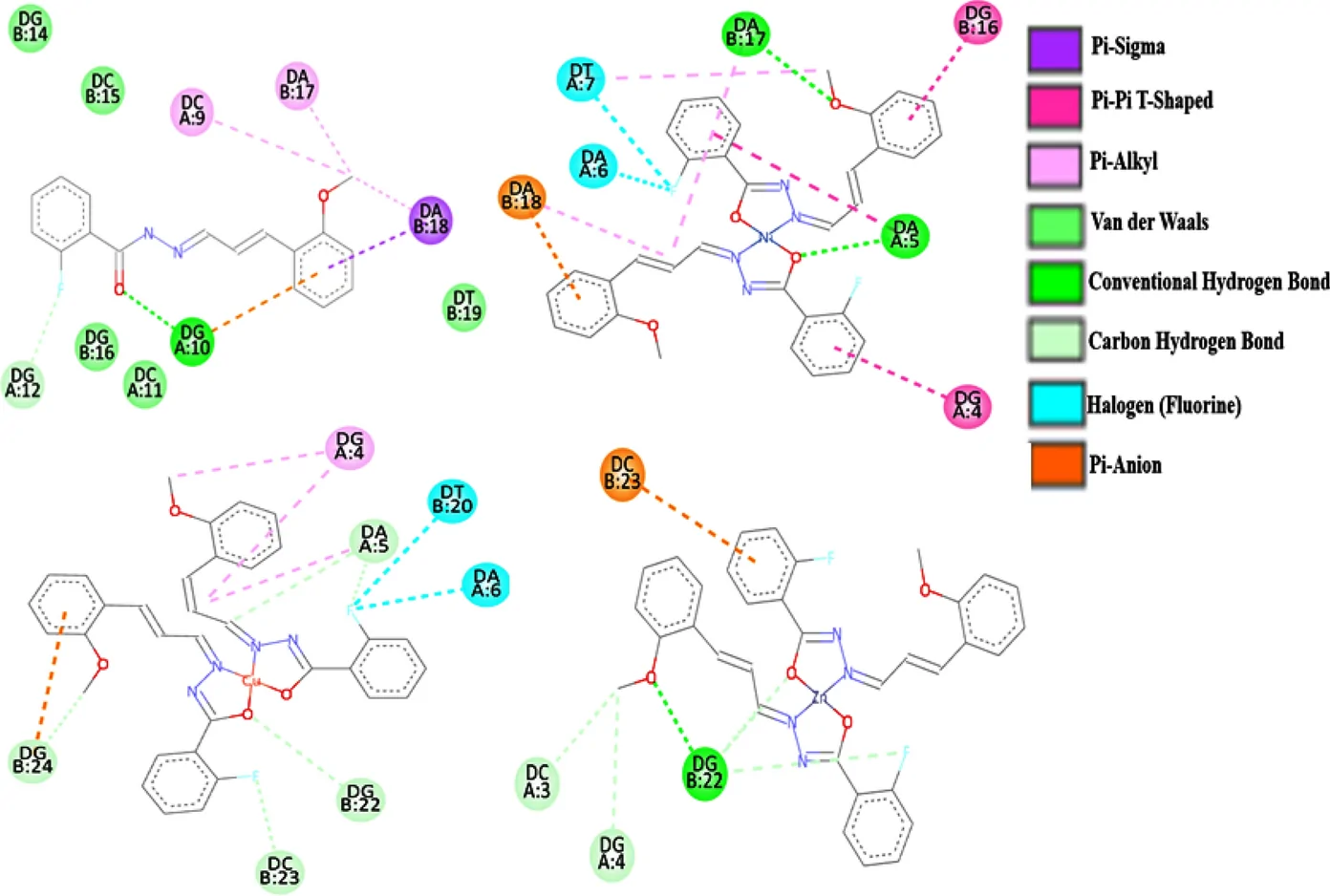
2D molecular docking interaction diagrams for the acyl-hydrazone ligand (L) and its metal complexes with Ni(II), Cu(II), and Zn(II) bound to calf thymus DNA (CT-DNA). The panels illustrate distinct non-covalent interactions, including π–stacking, halogen bonding, hydrogen bonds, and hydrophobic contacts supporting the proposed groove-binding mode. DNA bases and interaction types are labeled according to standard notation

diffusion-controlled limit (~ 10^1^⁰ M^−1^ s^−1^) (Paliwal et al. 2023; Parsckar et al. 2022). This suggests the possible formation of a ground-state complex between BSA and the compounds. However, in the absence of temperature-dependent fluorescence lifetime or UV–Vis absorption studies, a definitive distinction between static and dynamic quenching cannot be established. Nevertheless, the observed fluorescence quenching clearly confirms a strong interaction between the compounds and BSA. Figure 9ii (a) represents the Stern–Volmer plots, and (b) represents the double-logarithmic plots of [log(F_0_-F)/F vs. log[Q]], used to determine the binding constant (K_b_) and the number of binding sites (n). The analysis indicates a single independent binding site for the compounds on BSA. The calculated binding constant (K_b_) and Stern–Volmer constant (K_sv_) values for the ligand and its metal complexes were in the ranges of 10^4^–10^5^ M^−1^ and 10^5^ M^−1^, respectively. These values are consistent with moderate protein–ligand interactions commonly reported for serum albumin binding systems (K_b_ = 10^4^–10⁶ M^−1^ and K_sv_ = 10^2^–10⁸ M^−1^), (Smolková et al. 2023). Accordingly, the results indicate interaction between the compounds and BSA, which may influence their binding and transport behavior in biological environments.

### Molecular docking

Molecular docking simulations, Fig. 10, were performed to complement the experimental findings on the interaction between CT-DNA and the synthesized ligand, which is 2-fluoro-N′-[(1*E*,2*E*)-3-(2-methoxyphenyl)prop-2-en-1-ylidene]benzohydrazide, and its Ni(II), Cu(II), and Zn(II) complexes. Since an experimentally determined crystal structure of CT-DNA is unavailable, a canonical double-stranded B-DNA model was employed to represent the biologically relevant helical conformation of genomic DNA. The docking analysis revealed that the free ligand binds stably within the minor groove of DNA with a calculated binding affinity of –8.1 kcal mol^−1^. The interaction map shows two directional hydrogen bonds formed between the carbonyl oxygen and residues DG(A:10) (1.85 Å) and DG(B:16) (3.50 Å), along with a weak C–F···H hydrogen interaction with DG(A:12). These contacts, combined with π–anion (DG(A:10)) and π–alkyl (DA(B:17–18)) interactions, stabilize the ligand through a combination of polar and hydrophobic forces without distorting the DNA helix. The overall pattern is characteristic of minor-groove binding, consistent with the moderate hypochromism (35.5%), absence of significant bathochromic shift (Δλ < 5 nm), and the experimental K_b_ = 2.91 × 10^4^ L mol^−1^ obtained by UV–Vis titration(Ejidike et al. 2025, 2024).

The Ni(II) complex displayed one of the most favorable docking scores within the series (− 8.6 kcal mol^−1^). However, given the small differences among the calculated energies, these values should be interpreted qualitatively and primarily as evidence that the proposed binding pose is energetically feasible. The interaction diagram reveals two conventional hydrogen bonds between the coordinated carbonyl oxygen and residues DA(A:5) and DA(B:17) (2.5–3.0 Å), along with two halogen contacts (F···H-DA(A:6) and DT(A:7)) typical of groove-binding geometry. Additionally, π–anion and π–π T-shaped interactions with DA(B:18) and DG(A:4) stabilize the complex along the DNA groove. These multiple directional and hydrophobic interactions reinforce the non-intercalative, groove-binding mode, correlating well with the highest experimental binding constant (K_b_ = 5.07 × 10^4^ L mol^−1^) and the pronounced hypochromism (41.0%).

The Cu(II) complex interacts through a combination of three C–H···O hydrogen bonds (DA(A:5), DG(B:22), DC(B:23)) and two halogen bonds (DA(A:6), DT(B:20)) at distances of 2.7–3.1 Å. π–anion (DG(B:24)) and π-alkyl (DG(A:4)) contacts further stabilize the system. The geometry confirms that the complex anchors laterally along the minor groove without insertion between base pairs, consistent with a groove-binding mechanism. The docking score (–8.4 kcal mol^−1^) and experimental data (K_b_ = 3.59 × 10^4^ L mol^−1^; hypochromism = 28.9%) support this conclusion.

The Zn(II) complex displays binding affinity similar to the other complexes (–8.0 kcal mol^−1^) and a pattern dominated by hydrogen bonds (DC(A:3), DG(B:22)), a weak C–H···O contact (DG(A:4)), one π–anion (DC(B:23)), and one π–alkyl (DG(B:22)) interaction. These results confirm a groove-binding mode, with the Zn(II) center inducing local polarization but without intercalation. The slightly weaker theoretical and experimental affinities (K_b_ = 1.70 × 10^4^ L mol^−1^) indicate a more rigid, less hydrophobic interface than observed for Ni(II) and Cu(II).

The combined experimental and theoretical results indicate that the ligand and its metal complexes interact with CT-DNA predominantly through a non-intercalative minor-groove binding mechanism. The docking studies provided plausible and energetically favorable binding poses for all compounds, supporting the interaction patterns observed experimentally. Although the docking scores should be interpreted qualitatively, the proposed binding models are consistent with the UV–Vis, viscosity, and ethidium bromide displacement studies.

The supplementary material (Figure S3a) provides the three-dimensional docking representations for the ligand and its Ni(II), Cu(II), and Zn(II) complexes bound to CT-DNA. These 3D models complement the 2D interaction diagrams shown in Fig. 10, offering a spatial visualization of the groove-binding mode and the overall fit of each compound within the DNA minor groove.

## Conclusion

A previously unreported acylhydrazone Schiff base ligand, 2-fluoro-N′-[(1E,2E)-3-(2-methoxyphenyl)prop-2-en-1-ylidene]benzohydrazide (L), was synthesized, and its coordination chemistry toward Ni(II), Cu(II), and Zn(II) ions was systematically established. Comprehensive characterization confirmed that the ligand undergoes enolization and deprotonation upon complexation, acting as an N,O-bidentate chelator and forming thermally stable [M(L)₂] complexes (M = Ni, Cu, Zn) with well-defined polycrystalline domains. TGA–DSC, elemental analysis, EDTA titration, and HRESIMS validated the 1:2 metal-to-ligand stoichiometry.

Spectroscopic investigations demonstrated that metal coordination enhances π-delocalization and promotes ligand-to-metal electronic communication, particularly for Ni(II) and Cu(II). Biological interaction studies showed that all compounds bind to CT-DNA predominantly through a non-intercalative minor-groove mechanism, supported by moderate hypochromism, viscosity increase without helix distortion, EB displacement assays, and docking simulations. The DNA-binding studies suggested that the metal center influences the interaction with DNA, with the Ni(II) complex showing the strongest response among the compounds studied.

Furthermore, the BSA-binding experiments suggested a predominant static quenching component, indicating the possible formation of ground-state complexes with serum albumin. The obtained binding constants fall within the range typically associated with moderate and reversible protein–ligand interactions, suggesting potential relevance to protein-binding behavior in biological systems. The consistency between experimental observations and molecular docking results supports the proposed binding interactions and reinforces the reliability of the structural model.

Conclusively, this work presents a new acylhydrazone Schiff base scaffold and demonstrates that coordination with first-row transition metals significantly influences structural, electronic, thermal, and biomolecular interaction properties. These findings provide valuable structure–activity relationship insights for Schiff base metal complexes. Importantly, the Ni(II) complex emerges as the most promising candidate for further investigation due to its superior DNA and BSA binding performance. Future studies should focus on in vivo biological evaluation, detailed mechanistic investigations, and pharmacokinetic profiling to fully assess the therapeutic potential of this ligand system and its metal complexes.

## Supplementary Information

Below is the link to the electronic supplementary material.

Supplementary file1 (DOCX 755 kb)

## Data Availability

The datasets generated and/or analyzed during the current study are available from the corresponding author upon reasonable request.
