# Supplementary material for: Synthesis, characterization, and DNA binding of Ni(II), Cu(II), and Zn(II) complexes with a novel N,O-bidentate acylhydrazone
Source: Biometals. 2026 Jul 18;39(5):2135–58. doi: 10.1007/s10534-026-00856-0 (PMC13518446; doi:10.1007/s10534-026-00856-0)
Supplement: Supplementary file 1 [file 10534_2026_856_MOESM1_ESM.docx]

**Supplementary material**

**Synthesis, Characterization, and DNA Binding of Ni(II), Cu(II), and Zn(II) Complexes with a Novel N,O-Bidentate Acylhydrazone**

Aujenus Albert Msumange^a^*, Jeniffer Meyer Moreira^a^, Deus Albert Msumange^b^, Rafael Aparecido Carvalho Souza^a^, Mário Machado Martins^c^, [Cristiane Storck Schwalm](http://buscatextual.cnpq.br/buscatextual/visualizacv.do?id=K4537490A6)^a^, Luana da Silva Dorneles^a^, Magno Aparecido Gonçalves Trindade^a^, Raphael Rodrigues^a^, Eduardo José de Arruda^a^, Cláudio Teodoro de Carvalho^a^*

*^a^Federal University of Grande Dourados - Quality Control and Thermal Analysis Laboratory (LabCAT), Dourados, MS 79804-970, Brazil*

*^b^Centre for Advanced Materials and Industrial Chemistry (CAMIC), School of Science, RMIT University, Melbourne, VIC 3000, Australia*

*^c^Federal University of Uberlândia, Uberlândia, MG, 38400-902, Brazil*

**Corresponding author’s e-mail:** claudiocarvalho@ufgd.edu.br

**Purity analysis by DSC**

The DSC curve of the ligand (L), Figure S1, exhibits a distinct and well-defined endothermic peak at 205.6 °C, corresponding to its melting transition. The sharp and symmetrical nature of the peak indicates a highly crystalline and pure material. Based on van’t Hoff analysis using the Universal Analysis software (TA Instruments), the estimated purity of the sample is approximately 99.9%.

**Fig. S1** DSC curve of the ligand (L) for melting point and purity analysis

**High-resolution electrospray ionization mass spectrometry (HRESIMS)**

The compounds were analyzed by LC–MS, and the mass spectra of the metal complexes exhibit well-defined isotopic patterns arising from the natural isotopic distributions of Ni, Cu, and Zn. These characteristic isotope envelopes are consistent with the proposed metal–ligand stoichiometry and support the formation of the corresponding coordination complexes. The protonated ligand ion was observed at *m/z* 299.1188 [M + H]^+^ (calculated for C_17_H_16_FN_2_O_2_, 299.1190, Δ -0.67 ppm). The charged complexes ions were observed at *m/z* 653.1536 [M – H]^+^ [Ni(L)_2_], *m/z* 658.1476 [M – H]^+^ [Cu(L)_2_] and *m/z* 659.1425 [M – H]^+^ [Zn(L)_2_], in agreement with calculated values for C_34_H_29_F_2_N_4_O_4_Ni (653.1505, Δ 4.74 ppm), C_34_H_29_F_2_N_4_O_4_Cu (658.1448,
Δ +4.25 ppm) and C_34_H_29_F_2_N_4_O_4_Zn (659.1443, Δ -2.73 ppm), respectively.

**Fig. S2(a)** ESI-MS of ligand. MS (ESI, positive mode): [M + H]^+^ calculated for C_17_H_16_FN_2_O_2_, 299.1190, found: 299.1188, Δ -0.67 ppm

**Fig. S2(b)** ESI-MS of Ni(II) complex. MS (ESI, positive mode): [M – H]^+^ calculated for C_34_H_29_F_2_N_4_O_4_Ni, 653.1505, found: 653.1536, Δ +4.74 ppm

**Fig. S2(c)** ESI-MS of Cu(II) complex. MS (ESI, positive mode): [M – H]^+^ calculated for C_34_H_29_F_2_N_4_O_4_Cu, 658.1448, found: 658.1476, Δ + 4.25 ppm

**Fig. S2(d)** ESI-MS of Zn(II) complex. MS (ESI, positive mode): [M – H]^+^ calculated for C_34_H_29_F_2_N_4_O_4_Zn, 659.1443, found: 659.1425, Δ -2.73 ppm

**Molecular Docking (3D)**

Interaction studies between the acylhydrazone ligand and its metal complexes with DNA revealed distinct modes of recognition and anchoring to the double helix, with the minor groove emerging as the primary binding site (Figure S3). The free ligand exhibited multiple stabilizing interactions involving residues such as DG10, DG12, DC9, DA17, and DA18, characterized by hydrogen bonds and electrostatic contacts with phosphate groups, in addition to π–alkyl and π–σ hydrophobic interactions that promote aromatic packing. In the metal complexes, a similar behavior was observed, but with greater diversity and strength of interactions due to the presence of the metallic center. The nickel complex presented a balanced network of hydrogen bonds, halogen interactions, and π–π stacking with residues such as DA5, DA6, DT7, and DG16, indicating robust stabilization within the minor groove. The copper complex displayed a multifaceted interaction profile, with hydrogen and halogen bonds, as well as π–anion and hydrophobic interactions involving DA5, DG4, and DG24, suggesting a strong affinity for DNA and a cooperative mechanism between polar and dispersive forces. The zinc complex demonstrated strong electrostatic coupling with phosphate groups and multiple hydrogen bonds with DG22, DC3, and DC23, combined with π–π stacking with DG4, reinforcing its anchoring and selectivity.

Altogether, the results shown in Figure S3 indicate that the ligand and its metal complexes preferentially interact through the minor groove of DNA, stabilized by a combination of hydrophobic, halogen, hydrogen, and electrostatic forces, conferring them selective molecular recognition potential and relevant biological activity.

**Fig. S3a** Three-dimensional molecular docking models showing the interaction of the free ligand (L) and its Ni(II), Cu(II), and Zn(II) complexes with DNA. All systems indicate a minor groove-binding mode, with the ligands aligned parallel to the helical axis and stabilized by hydrogen bonds, π–π/π–alkyl, and halogen (F···H) interactions

Before the docking analysis of the metal complexes, the reliability of the docking protocol was evaluated through a redocking experiment using the co-crystallized ligand of the 4U8A structure. As shown in Figure S3b, the predicted pose exhibited an excellent overlap with the experimental crystallographic conformation, yielding a Heavy Atom RMSD value of 0.593 Å. Since this value is well below the commonly accepted validation threshold of 2.0 Å, the docking protocol was considered suitable for reproducing the experimentally observed binding mode. Although no crystallographic DNA–complex structures are available for direct comparison, the successful recovery of the native ligand pose increases confidence in the binding orientations predicted for the Ni(II), Cu(II), and Zn(II) complexes. Therefore, the minor groove-binding modes observed for all systems in Figure S3a can be regarded as structurally plausible within the limitations of the molecular docking approach.

**Figure S3b** Overlap of the co-crystallized native ligand and the reattached ligand
